# Spherical Wave Propagation in a Poroelastic Medium with Infinite Permeability: Time Domain Solution

**DOI:** 10.1155/2014/813097

**Published:** 2014-02-18

**Authors:** Mehmet Ozyazicioglu

**Affiliations:** Department of Civil Engineering, Ataturk University, 25240 Erzurum, Turkey

## Abstract

Exact time domain solutions for displacement and porepressure are derived for waves emanating from a pressurized spherical cavity, in an infinitely permeable poroelastic medium with a permeable boundary. Cases for blast and exponentially decaying step pulse loadings are considered; letter case, in the limit as decay constant goes to zero, also covers the step (uniform) pressure. Solutions clearly show the propagation of the second (slow) *p*-wave. Furthermore, Biot modulus *Q* is shown to have a pronounced influence on wave propagation characteristics in poroelastic media. Results are compared with solutions in classical elasticity theory.

## 1. Introduction 

Cavity pressurization problems (circular and spherical) constitute one of the basic problems of wave mechanics and since early 1930s considerable scientific work has been published on cavity problems in classical elasticity theory [1–5]. These problems generally are amenable to exact solution and the analytical solutions cast new light onto the nature of wave propagation in solid media. The problem has practical applications in geophysics, seismology, and, tunnel and mining engineering, like earthquake sources, underground detonation and seismic probing. The exact solutions of such simple problems serve to understand more complex wave motions; moreover, these solutions can also be used as benchmark problems to assess the accuracy of numerical methods (FEM, BEM, FDM, etc.) [6]. Being a relatively new extension of classical elasticity theory, the corresponding work (exact time or frequency domain solutions) in poroelasticity is rare. Notable efforts are the Laplace domain solution of circular cavity problem [7, 8] and frequency domain solution of suddenly pressurized spherical cavity [9]; an analytical solution in Laplace domain for a dynamically loaded poroelastic column [10, 11] is also available. Fundamental solutions of poroelastodynamics can be found in [12–15]. Solution of Lamb's Problem in poroelastic half space is given by Philippacopoulos [16]. The reader is referred to the review article [17] for a compendium of other analytical and numerical solutions.

This work concerns time domain analytical solution of dynamic pressurization of a spherical cavity in an infinite poroelastic medium with permeable boundary and quiescent initial conditions. Time domain solutions are derived by analytical inverse Fourier Transform using complex residue theorem. Since finite permeability renders frequency domain equations extremely difficult to invert, infinite permeability is assumed in the medium. Infinite permeability is a reasonable approximation for coarsely grained media like gravely soils or pebbles. The analytical solutions are derived for Dirac (blast), exponentially decaying step pulse as well as constant uniform (Heaviside) pressure. The developed solutions clearly show the existence of a second pressure wave, the so-called slow wave.

### 1.1. Biot's Theory of Poroelasticity

Unlike the classical elasticity theory, Biot's theory of poroelasticity is a coupled deformation-flow theory of a porous solid matrix with interstitial fluid. Biot introduced his linear quasistatic theory in 1941 [18] and later extended it to cover the dynamic range [19, 20]. An extensive review of quasistatic poroelasticity can be found in [21, 22].

The constitutive equations of linear-isotropic poroelasticity are (summation convention applies)  

(1a) $$\begin{array}{c} \tau_{ij}=2\mu \varepsilon_{ij}+\lambda \delta_{ij}\varepsilon_{kk}-\alpha \delta_{ij}p, \end{array}$$

(1b) $$\begin{array}{c} \theta =\alpha \varepsilon_{kk}+\frac{1}{Q}p, \end{array}$$

where *ε*_*ij*_ = (1/2)(*u*_*i*,*j*_ + *u*_*j*,*i*_) are the strains in the solid *u*_*i*_ and *τ*_*ij*_ are components of solid displacement vector and total stress tensor, *p* is the fluid pressure, *θ* is the variation of fluid volume per unit reference volume, and *δ*_*ij*_ is the Kronecker delta. Here, tensile *τ*_*ij*_ and *ε*_*ij*_ are positive, while pore pressure *p* is positive when being compressive. The four material constants of poroelastic media are

 
*λ*: drained Lame's modulus, as defined in classical elasticity theory (dimension *≡* F/L^2^),
 
*μ*: drained shear modulus, as defined in classical elasticity theory (dimension *≡* F/L^2^),
 
*α*: Biot's effective stress coefficient (dimensionless),
 
*Q*: Biot deformation modulus, corresponding to the reciprocal of constrained storage coefficient in hydrogeology (dimension *≡* F/L^2^).

## 2. Governing Equations for Spherical Symmetry

**Figure 1:**
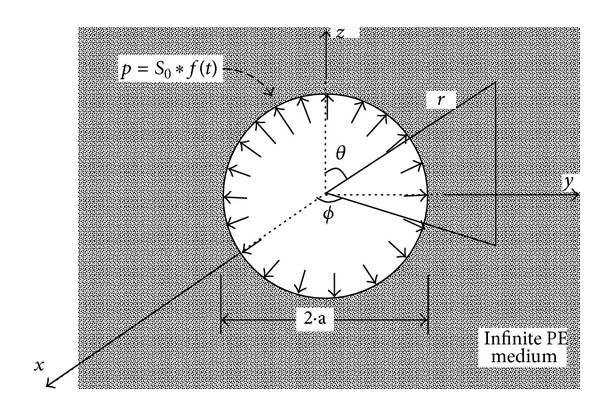
Description of problem, pressurized spherical cavity in infinite poroelastic medium.

Consider a spherical cavity of radius “*a*” in an infinite PE medium (Figure 1). Let *u*_*r*_, *u*_*ϕ*_, and *u*_*θ*_ be the spherical components of solid displacements. Because the cavity is spherically symmetric and we assume a time varying but spherically symmetric pressure inside, waves emanating from such a source will have spherical symmetry; that is,

(2) $$\begin{array}{c} u_{r}=u\left(r,t\right)\neq 0,\\ u_{\theta }=u_{\phi }=\frac{\partial }{\partial \theta }\left(\cdot \right)=\frac{\partial }{\partial \phi }\left(\cdot \right)=0. \end{array}$$

The Fourier Transform and its inverse on time variables are defined as

(3) uF(ω) =∫−∞∞u(t)e−iωtdt⟷u(t)=12π∫−∞∞uF(ω)eiωtdω.

The governing equations of 3D poroelasticity in Fourier Transform Space (FTS) or frequency domain in this case reduce to the following (the reader is referred to [9] for derivation):

(4) (λ+2μ)[1r2ddr(r2duFdr)−2r2uF]  −(α+β)dpFdr+ω2(ρ+βρf)uF=0,−βω2ρf1r2ddr(r2dpFdr)+(α+β)1r2ddr(r2uF)  +pFQ=0.

Here, *u* denotes the only nonzero displacement for simplicity, that is, radial component *u*_*r*_, *ω* is frequency, and *β* is a coefficient defined as

(5) $$\begin{array}{c} \beta =\frac{n^{2}\kappa \rho_{f}\omega^{2}}{i\omega n^{2}-\omega^{2}\kappa \left(\rho_{a}+n\rho_{f}\right)}. \end{array}$$

The stress components are related to the radial displacement *u* as

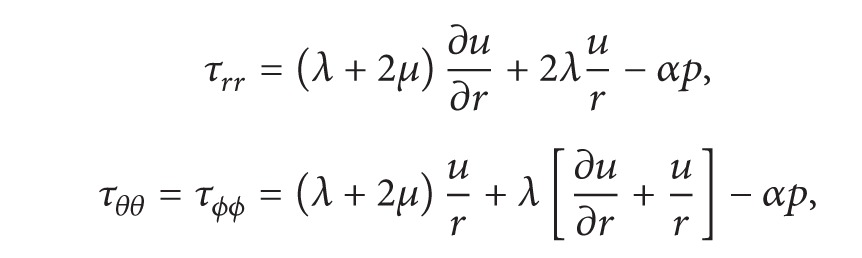
(6)

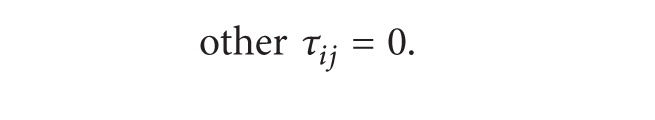
(7)


### 2.1. Boundary Conditions

Among various possible combinations of traction, displacement, pore pressure, and fluid flux, only the permeable boundary condition will be considered here; that is,

(8) $$\begin{array}{c} {\tau_{rr}|}_{r=a}=-S_{0}f\left(t\right),\\ {p|}_{\;r=a}=0, \end{array}$$

where *S*_0_ is the amplitude (strength) of the force and *f*(*t*) is the time variation of the stress on the boundary, and here, two cases will be considered:

(9) $$\begin{array}{c} f\left(t\right)=\{\begin{array}{cc} \delta \left(t\right), & \text{Dirac}\;\;\text{pulse},\\ e^{-\gamma t}H\left(t\right), & \text{exponentially}\;\;\text{decaying}\;\;\text{step}\;\;\text{pulse}, \end{array} \end{array}$$

where *γ* is decay constant. It is clear that when decay constant “*γ*” is taken to be zero, the second boundary condition reduces to step load (Heaviside) boundary condition. Thus, the solution for latter boundary condition will also include the step (Heaviside) pressure as a special case.

### 2.2. Analytical Solution in Frequency Domain

The complete analytical solution of this problem in frequency domain [9] is

(10) uF=aS0F(ω)Δ×[(m22ω2+λ42)[1r+iωm1]e−iωm1(r−a)r  −(m12ω2+λ42)[1r+iωm2]e−iωm2(r−a)r],pF=λ+2μα+βaS0rF(ω)(m22ω2+λ42)(m12ω2+λ42)Δ×[e−iωm1(r−a)−e−iωm2(r−a)],

where

(11) Δ=[4μa(iωm1+1a)−(λ+2μ)ω2m1](m22ω2+λ42)−[4μa(iωm2+1a)−(λ+2μ)ω2m2](m12ω2+λ42),

(12) $$\begin{array}{c} F\left(\omega \right)=\int_{-\infty }^{\infty }f\left(t\right)e^{-i\omega t}dt, \end{array}$$

where

(13) $$\begin{array}{c} \lambda_{4}^{2}=-\omega^{2}{\overset{-}{\lambda }}_{4},\\ m_{1}=\sqrt{\frac{1}{2}\left[B-\sqrt{B^{2}+4C}\right]},\\ m_{2}=\sqrt{\frac{1}{2}\left[B+\sqrt{B^{2}+4C}\right]},\\ {\overset{-}{\lambda }}_{4}^{2}=\frac{\left(\rho +\beta \rho_{f}\right)}{\lambda +2\mu },\\ B=\frac{\rho +\beta \rho_{f}}{\lambda +2\mu }-\frac{\rho_{f}}{\beta Q}-\frac{\rho_{f}{\left(\alpha +\beta \right)}^{2}}{\beta \left(\lambda +2\mu \right)},\\ C=\frac{\rho_{f}\left(\rho +\beta \rho_{f}\right)}{\beta Q\left(\lambda +2\mu \right)}. \end{array}$$

Stresses can be found by inserting (10) in (6). The two terms in each of (10) represent two *p*-wave phases with different propagation characteristics. The reciprocal of real part of *m*_1_ and *m*_2_ correspond to propagation velocities of fast and slow (second) longitudinal waves. The second (slow) *p*-wave is highly dispersive (frequency dependent) and highly damped; dispersion relation is given in [9].

## 3. Exact Time Domain Solution 

Exact inversion of (10) for time domain solutions is very difficult if not impossible; this difficulty arises from frequency dependence of wave slowness terms (*m*_1_, *m*_2_). This dependence can be removed by assuming infinite permeability in the medium. In this case (*κ* → *∞*), the coefficient *β* becomes

(14) $$\begin{array}{c} \beta =-\frac{n^{2}\rho_{f}}{\rho_{a}+n\rho_{f}} \end{array}$$

and thus, *m*_1_, *m*_2_ become free of *ω* and both waves become nondispersive.

In this case, the discriminant Δ in (11) can be factored as

(15) $$\begin{array}{c} \Delta =\left(\lambda +2\mu \right)\left(m_{1}^{2}-m_{2}^{2}\right){\overset{-}{\lambda }}_{4}^{2}\left(\omega -\Omega_{1}\right)\left(\omega -\Omega_{2}\right)\omega^{2}, \end{array}$$

where

(16) $$\begin{array}{c} \Omega_{1}=i\Gamma +N,\;\;\Omega_{2}=i\Gamma -N,\\ \Gamma =\frac{2\mu }{\left(\lambda +2\mu \right)a{\overset{-}{\lambda }}_{4}^{2}}\frac{\left(m_{1}m_{2}+{\overset{-}{\lambda }}_{4}^{2}\right)}{m_{1}+m_{2}},\\ N=\frac{2\mu }{\left(\lambda +2\mu \right)a{\overset{-}{\lambda }}_{4}^{2}}\sqrt{\frac{\lambda +2\mu }{\mu }{\overset{-}{\lambda }}_{4}^{2}-{\left[\frac{\left(m_{1}m_{2}+{\overset{-}{\lambda }}_{4}^{2}\right)}{m_{1}+m_{2}}\right]}^{2}}. \end{array}$$

It can be shown that both Γ and *N* are positive constants. Thus, (10) can now be written as follows.

### 3.1. Frequency Domain Solution

(17) uF=aS0F(ω)(λ+2μ)(m12−m22)λ−42  r×  [(m22−λ−42)[1/r+iωm1]e−iωm1(r−a)(ω−Ω1)(ω−Ω2)  −(m12−λ−42)[1/r+iωm2]e−iωm2(r−a)(ω−Ω1)(ω−Ω2)],pF=aS0(m22−λ42)(m12−λ42)(α+β)(m12−m22)λ−42rF(ω)(ω−Ω1)(ω−Ω2)×[e−iωm1(r−a)−e−iωm2(r−a)].

### 3.2. Time Domain Solution

The general form of time domain solutions is obtained by inverse Fourier Transform; by (3),

(18) u(t,r)=aS02π(λ+2μ)(m12−m22)λ−42r×∫−∞∞[(m22−λ−42)[1/r+iωm1]F(ω)eiωτ1(ω−Ω1)(ω−Ω2)    −(m12−λ−42)    ×[1/r+iωm2]F(ω)eiωτ2(ω−Ω1)(ω−Ω2)]dω,p(t,r)=aS0(m22−λ42)(m12−λ42)2π(α+β)(m12−m22)λ−42r×∫−∞∞F(ω)(ω−Ω1)(ω−Ω2)[eiωτ1−eiωτ2]dω,

where *τ*_*k*_ = *t* − *m*_*k*_(*r* − *a*), *k* = 1,2. The two integrals pertain to the two *p*-waves in theory of poroelasticity.

Depending on the boundary pressures, the denominators in (18) have the following simple poles:

 (i) Dirac pulse
 
*F*(*ω*) = 1, simple poles at *Ω*_1_, *Ω*_2_,
 (ii) exponentially decaying step load,
 
*F*(*ω*) = 1/*γ* + *iω*, poles at *Ω*_1_, *Ω*_2_, and *iγ*.

**Figure 2:**
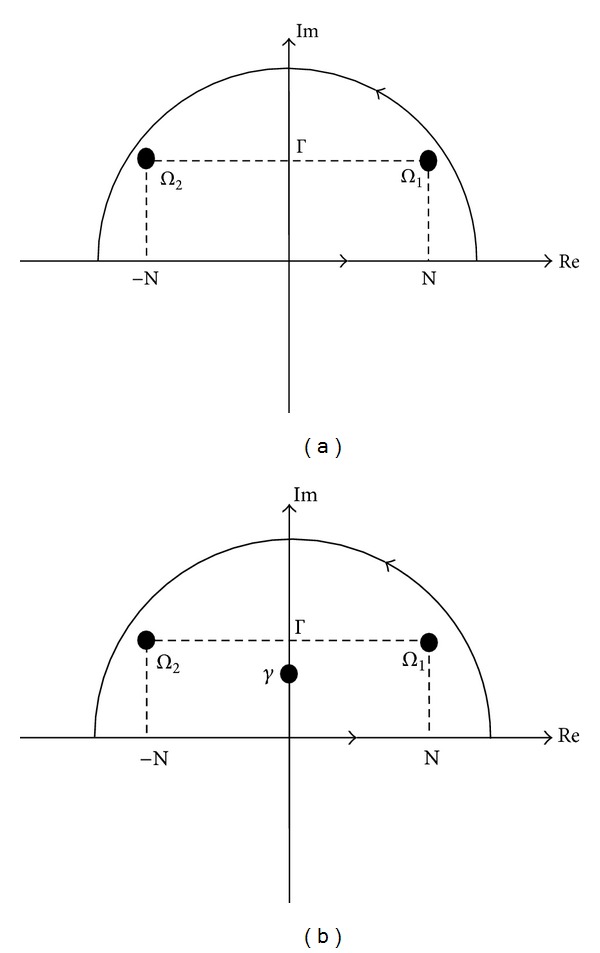
Contours of integration and position of poles in the complex plane. (a) Dirac pulse. (b) Exponentially decaying step pulse.

Solution for Heaviside step pulse (pressure applied and maintained) is obtained in the limit *γ* → 0 from the second case. Since the constants *N*, *γ* are positive, all poles are in the upper half plane (Figure 2). For *τ* > 0, the integral is evaluated along contour above the real axis (Figure 2), yet when *τ* < 0, the contour below real axis gives zero.

Define

(19) $$\begin{array}{c} \overset{-}{A}=\frac{-a\cdot S_{0}}{\left(\lambda +2\mu \right){\overset{-}{\lambda }}_{4}^{2}\left({m_{1}^{2}}^{}-{m_{2}^{2}}^{}\right)\cdot N\cdot r},\\ \overset{-}{B}={m_{2}^{2}}^{}-{\overset{-}{\lambda }}_{4}^{2},\\ \overset{-}{C}={m_{1}^{2}}^{}-{\overset{-}{\lambda }}_{4}^{2}. \end{array}$$

The integrals in (18) along the countours in Figure 2 are evaluated by the complex residue theorem, the results are as follows:

(i) Dirac (blast) pressure:

(20) u(r,t)=A−[B−e−Γτ1{(1r−m1Γ)sin(Nτ1)     +m1Ncos⁡(Nτ1)}H(τ1)−C−e−Γτ2  ×{(1r−m2Γ)sin(Nτ2)+m2Ncos⁡(Nτ2)}  ×H(τ2)].

Porepressure wave:

(21) p(r,t)=(λ+2μ)A−·B−·C−(α+β)×[e−Γτ1{2ΓNcos⁡(Nτ1)+(N2−Γ2)sin(Nτ1)}  ×H(τ1)−e−Γτ2  ×{2ΓNcos⁡(Nτ2)+(N2−Γ2)sin(Nτ2)}  ×H(τ2)].

(ii) Exponentially decaying step pulse:

Radial displacement:

(22) u(r,t)=−A−·N{(Γ−γ)2+N2}·[B−·G1(r,t)·H(τ1)−C−·G2(r,t)·H(τ2)],

where

(23) Gi(r,t)=(γ·mi−1r)·e−γτi−e−ΓτiN[(γ·mi−1r)·N·cos⁡(Nτi)    +[miΓ(Γ−γ)+miN2−Γ−γr]    ·sin(Nτi)].

Porepressure:

(24) p(r,t)=−(λ+2μ)·A−·B−·C−(α+β)×[f(τ1)H(τ1)−f(τ2)H(τ2)],

where

(25) f(x)=γ2e−γ·x(Γ−γ)2+N2+e−Γ·x2N{(Γ−γ)2+N2}×[2N{2Γ(Γ−γ)+(N2−Γ2)}cos⁡(N·x)  +2{(Γ−γ)(N2−Γ2)−2ΓN2}sin(N·x)].

(iii) Heaviside (step) pulse.

This solution can be obtained either by convolution in time domain of Heaviside function with impulse response function in (i) or by taking the limit *γ*→0 of (ii); both methods have been checked to give the same results:

Displacement:

(26) $$\begin{array}{c} u\left(r,t\right)=\overset{-}{A}\cdot \left[\overset{-}{B}\cdot F_{1}\left(r,t\right)-\overset{-}{C}\cdot F_{2}\left(r,t\right)\right]; \end{array}$$

where

(27) Fi(r,t)=H(τi)(Γ2+N2)·r×{N+e−Γτi[{mir(Γ2+N2)−Γ}      ×sin(Nτi)−Ncos⁡(Nτi)]}.

Porepressure:

(28) p(r,t)=−(λ+2μ)·A−·B−·C−(α+β)×[e−Γτ1{Ncos⁡(Nτ1)−Γsin(Nτ1)}H(τ1)  −e−Γτ2{Ncos⁡(Nτ2)−Γsin(Nτ2)}H(τ2)].

### 3.3. Solution in the Classical Theory of Elasticity

The elastodynamic solution in frequency domain for suddenly pressurized spherical cavity can be derived following a similar outline described above and it is found in [1–5, 23]. The solution given in Graff ([23] p. 298) does not satisfy the boundary condition at *t* = 0; the solution for exponentially decaying pressure is first derived by Blake [3], but explicit form is not given for radial displacement. Thus, the elastodynamic solutions that comply with the Fourier Transform definition in (10) are rederived. Here, we only state the results.

General solution for zero initial conditions $u\left(r,t=0\right)=0,\;\;\dot{u}\left(r,t=0\right)=0$ and boundary pressure *σ*_*r*_  (*r* = *a*, *t*) = −*p*(*t*), *σ*_*rϕ*_  (*r* = *a*, *t*) = 0,   *σ*_*rθ*_  (*r* = *a*, *t*) = 0:

(29) $$\begin{array}{c} u\left(r,t\right)=-\frac{a}{2\pi \cdot \rho_{e}\cdot r}\int_{-\infty }^{\infty }\frac{p\left(\omega \right)\left(i\cdot k+1/r\right)}{\left(\omega -\Omega_{1}\right)\left(\omega -\Omega_{2}\right)}e^{i\omega \tau }d\omega . \end{array}$$

(i) Dirac pulse (Blast loading):
  (30) ue(r,t)=a·S0Ne·ρe·re−Ne·τ×[(1r−Mec1)sin(Ne·τ)+Nec1cos⁡(Ne·τ)]·H(τ).
(ii) Heaviside:
  (31) ue(r,t)=a·S0(Me2+Ne2)·ρe·r×[1r−e−Me·τNe  ×{(Mer−Me2+Ne2c1)  sin(Ne·τ)    +Nercos⁡(Ne·τ)}]·H(τ).
(iii) Exponentially decaying step pulse:
  (32) ue(r,t)=a·S0((Me−γ)2+Ne2)·ρe·r×[(1r−γc1)·e−γ·τ−e−Me·τNe  ×{(Me−γr−Me(Me−γ)+Ne2c1)    ×sin(Ne·τ)    +Ne(1r−γc1)cos⁡(Ne·τ)}]·H(τ),
  where *u*_*e*_ is elastic radial displacement, *ρ*_*e*_ is the density of elastic medium, and $c_{1}=\sqrt{(\lambda +2\mu )/\rho_{e}}$, and $c_{2}=\sqrt{\mu /\rho_{e}}$ are the *p*- and *s*-wave velocities in an ideal elastic medium and
  (33) $$\begin{array}{c} k=\frac{\omega }{c_{1}},\;\;\eta^{2}=\frac{\lambda +2\mu }{4\mu },\\ M_{e}=\frac{c_{1}}{2\eta^{2}a},\;\;N_{e}=\frac{c_{1}}{2\eta^{2}a}\sqrt{4\eta^{2}-1},\\ \Omega_{1,2}=i\cdot M_{e}\mp N_{e},\;\;\tau =t-\frac{r-a}{c_{1}}. \end{array}$$
  Other parameters are as defined previously.

## 4. Results in Time Domain

**Figure 3:**
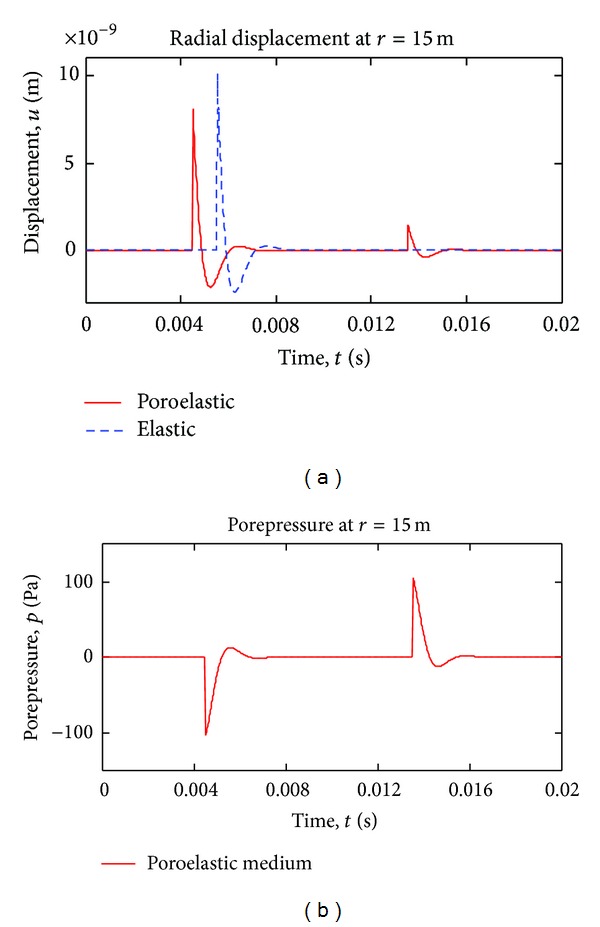
Time variation of radial displacement and pore pressure, blast (Dirac) pulse case.

**Figure 4:**
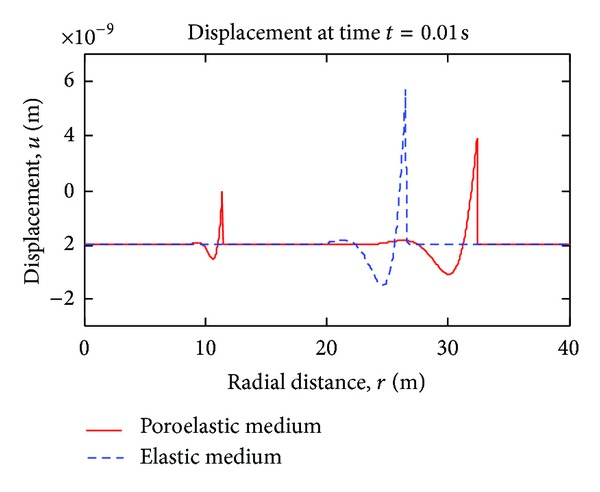
Displacement waves in elastic and poroelastic media (*t* = 0.01 s), blast (Dirac) pulse case.

**Figure 5:**
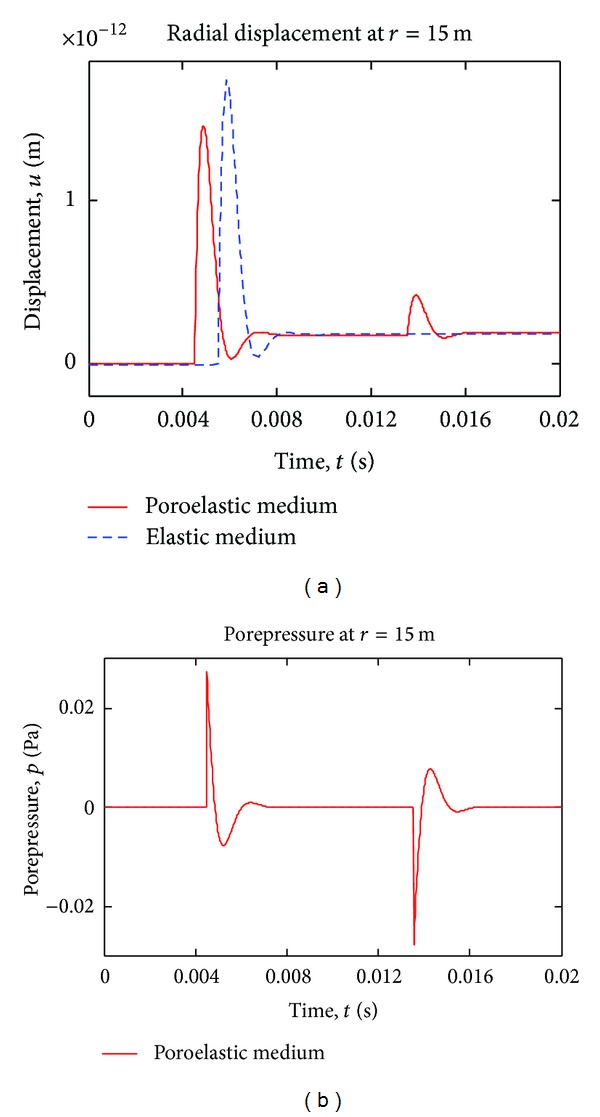
Time variation of radial displacement and pore pressure, step (Heaviside) pulse case.

**Figure 6:**
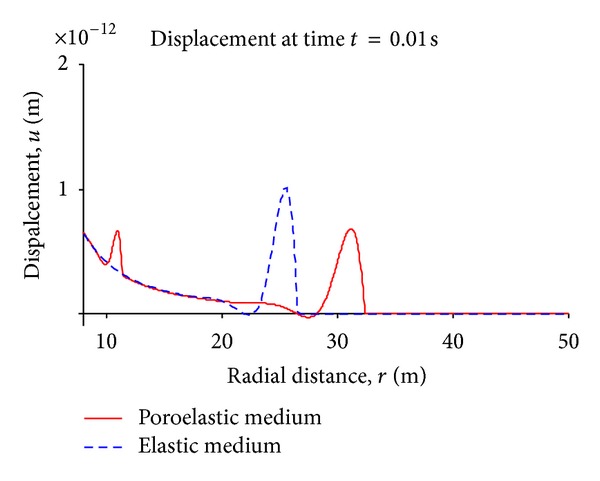
Displacement wave in the media at a particular time, step (Heaviside) pulse case.

**Figure 7:**
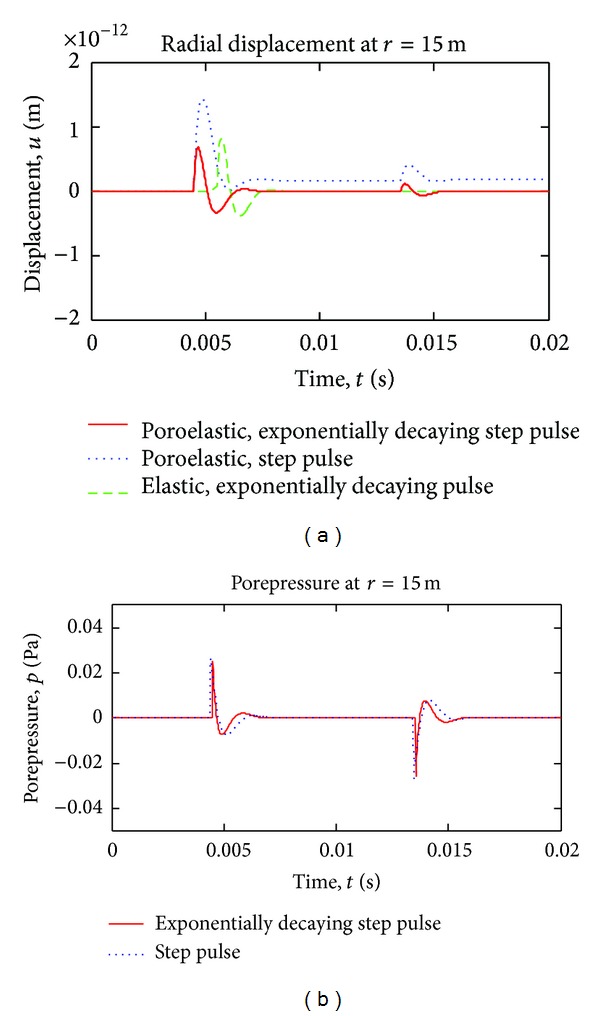
Time variation of radial displacement and pore pressure, exponentially decaying pulse case.

**Figure 8:**
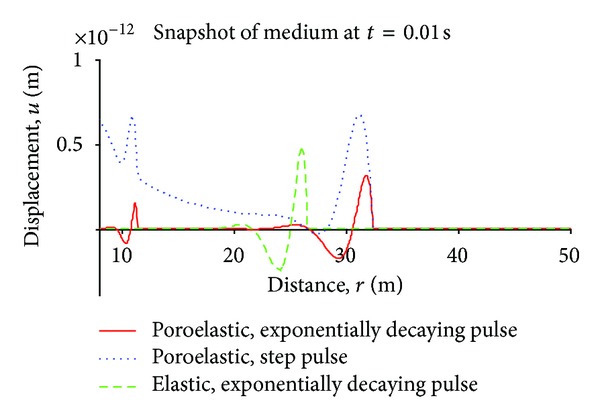
Displacement wave in the media at a particular time, exponentially decaying pulse case.

**Figure 9:**
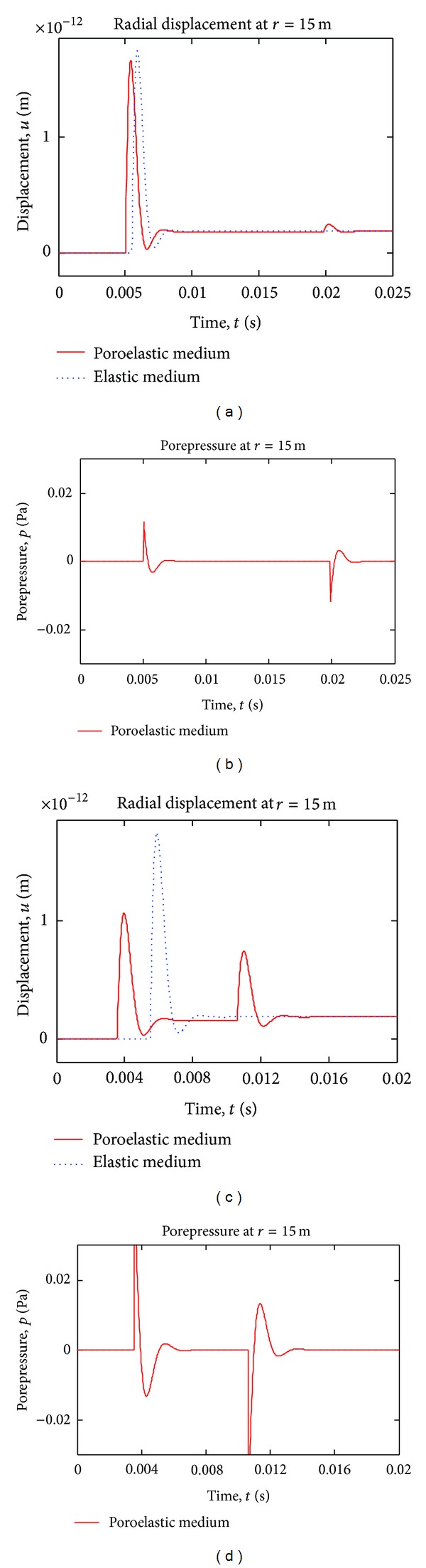
Time variation of radial displacement and pore pressure (step pulse case), for two different Biot moduli *Q*. ((a), (b)) are for *Q* = 0.5 × 10^10^ Pa; ((c), (d)) are for *Q* = 3.5∗10^10^ Pa.

Equations (20)–(32) are plotted for comparison and interpretation in Figures 3–9. In all of the computations, the material constants (Table 1) for Berea Sandstone [21, 22] are used, except for the permeability which is taken to be infinite.

The material parameters for corresponding elastic medium are as in Table 2.

These material constants give the *p*-wave velocities as the following:

 fast *p*-wave velocity: 1/*M*_1_ = 3137 m/s,
 slow *p*-wave velocity: 1/*M*_2_ = 1037 m/s,
 elastic *p*-wave velocity: *c*_1_ = 2551 m/s.

Figure 3 shows time variation of displacement and porepressure at station *r* = 15 m, for blast loading on the boundary of the cavity. The slow *p*-wave is clearly seen, as opposed to finite permeability case [9]. As seen from the figure, the primary wave in poroelastic medium arrives earlier than the elastic wave, and the slow poroelastic wave follows the latter with smaller amplitude. In finite permeability case [9], the slow *p*-wave in poroelastic material dies away very quickly, because of high dispersion and damping; thus, it can be said that the slow wave can be detected only in highly permeable media. The elastic wave has larger amplitude than the primary *p*-wave in Biot's theory, and this is because the energy is divided between primary and secondary waves in a poroelastic material. No permanent deformation (consolidation) is observed in the poroelastic medium under blast loading (pressure applied and removed).

Figure 4 is an alternative display of the variation of displacements at time *t* = 0.01 sec. with radial distance. As seen, the primary poroelastic wave (right most) leads the other two.

Figure 5 shows time variation of displacement and porepressure at station *r* = 15 m for step pulse boundary condition. It is seen that the primary wave in poroelastic medium arrives earlier than the elastic wave, with the slow poroelastic wave, having rather a smaller amplitude, following the latter. Again the elastic wave has larger amplitude than the primary *p*-wave in Biot's theory. Under step loading (pressure applied and maintained), a static deformation (consolidation) is observed to remain in the medium after both waves pass by the station.

Figure 6 is a snap shot of the the waves in the medium at time *t* = 0.01 sec. Again we observe permanent deformation forming behind the waves.

Figure 7 shows time variation of displacement and porepressure at station *r* = 15 m, for exponentially decaying boundary pressure case. In the figure, step pulse solution is also plotted for comparison. Although, a permanent deformation (consolidation) is observed under step loading, in exponentially decaying pulse case, the deformation is removed after the waves pass by the station completely, as expected, since the load goes to zero with time.

Figure 8 is a snap shot of the waves in the medium at time *t* = 0.01 sec. Step pulse case is included for comparison. The permanent deformation in the case of step pulse is clearly seen, while no permanent (static) deformation is observed in exponentially decaying step pulse case behind the two waves.

In some studies [24], Biot modulus *Q* is ignored for simplicity. In Figure 9, the effect of *Q* modulus is investigated for two different *Q* values.

The Biot deformation modulus *Q* has significant effect on wave propagation in porous media; as seen in Figure 9, reducing the Biot Modulus lowers wave speeds in poroelastic medium (compare to Figure 5) rendering fast p-wave get closer to the elastic wave and transfers energy of the slow wave to the fast wave; further reduction of Q results in the slow wave to disappear in the limit. Moreover, lower *Q* value causes porepressure wave to disappear as well, in the limit. On the other hand, high *Q* modulus increases poroelastic wave speeds compared to elastic wave and transfers more energy from fast wave to the slow wave; in this case (higher *Q*), the slow wave as well as porepressure wave amplitudes grow.

## 5. Conclusions

Time domain analytical solution of waves propagating from the surface of a pressurized spherical cavity in an infinitely permeable poroelastic medium are given for three types of boundary pressures (Dirac, Heaviside, and exponentially decaying step pulse). The problem is one of the classical problems in wave propagation in elastic solids [1, 23]. Among other possibilities of boundary conditions, this paper considers permeable boundary conditions; that is,  *p*(*r* = *a*, *t*) = 0.

Time domain solutions are obtained by calculating the residues in the inversion integral. The waves in infinitely permeable poroelastic medium are not dispersive, as opposed to finite permeability [9] case. Thus, it can be said that permeability governs the dispersion characteristics of waves in a poroelastic medium. The slow wave, clearly visualised in the graphics, has relatively small amplitude compared to fast wave, with the common material constants of Berea Sandstone.

It is also shown that Biot modulus has significant effect on the wave propagation speeds and amplitudes of both displacement and porepressure and thus should not be neglected in analysis or experimental measurements.

The results have an implication in underground sounding: since elastic *p*-wave is slower than poroelastic fast *p*-wave, current inversion methods, using classical elastic theory, based on measured velocities might be overestimating material moduli of earth materials.

## Figures and Tables

**Table 1:** Material data for Berea Sandstone (poroelastic medium).

*n*	*α*	*Q* (Pa)	*μ* (Pa)	*ν*	*κ* (m^4^/N/s)	*ρ* (kg/m^3^)	*ρ*_*f*_ (kg/m^3^)	*ρ*_*a*_ (kg/m^3^)
0.19	0.778	1.353∗10^10^	6∗10^9^	0.2	*∞*	2458	1000	125.4

**Table 2:** Material data for elastic medium.

*μ* (Pa)	*λ* (Pa)	*ν*	*ρ* (kg/m^3^)
6∗10^9^	4∗10^9^	0.2	2458
